# Comparative Evaluation of the Incidence of Dentin Microcracks Following Biomechanical Preparation Using Four Different Nickel-Titanium Rotary File Systems: An In Vitro Study

**DOI:** 10.7759/cureus.53506

**Published:** 2024-02-03

**Authors:** Rejin Mariyam, Navjot Singh Khurana, Jagvinder Singh Mann, Haridarshan Singh Sidhu, Sergy A, Mahesh Mohan

**Affiliations:** 1 Department of Conservative Dentistry and Endodontics, Government Dental College and Hospital, Patiala, IND; 2 Department of Pediatric and Preventive Dentistry, Government Dental College and Hospital, Patiala, IND; 3 Department of Conservative Dentistry and Endodontics, Institute of Dental Studies and Technologies, Ghaziabad, IND

**Keywords:** 2shape, trunatomy, stereomicroscope, protaper gold, neoendo flex, dentin microcracks

## Abstract

Introduction: Biomechanical preparation has gotten easier over time with the development of nickel-titanium (NiTi) rotary instruments. Despite their benefits, research has shown that these files frequently result in microcracks in the root canal dentin, which can fracture the roots. Such mishaps should be prevented, as they compromise the integrity of the root and reduce the long-term survival of endodontically treated teeth.

Materials and methods: This study was conducted at Government Dental College and Hospital, Patiala, Punjab, India. Eighty permanent mandibular premolar teeth were included. All the roots were inspected for any pre-existing cracks or craze lines under a stereomicroscope. The teeth were decoronated and then divided into four groups (n = 20): Group I: TruNatomy, Group II: Neoendo Flex, Group III: ProTaper Gold, and Group IV: 2Shape. The samples were instrumented according to the group to which they belonged. The roots were then sectioned horizontally at 3 mm and 6 mm from the apex and examined under a stereomicroscope at 40x for the presence of microcracks.

Results: The data were analyzed using the IBM SPSS Statistics for Windows, version 26 (released 2019; IBM Corp., Armonk, New York, United States). A chi-square test was applied, and the level of significance was set at p < 0.05. The highest incidence of microcracks was associated with ProTaper Gold (65%), followed by Neoendo Flex (45%), TruNatomy (20%), and 2Shape (20%).

Conclusion: All rotary instruments resulted in dentinal damage. ProTaper Gold exhibited the highest frequency of dentin cracks. TruNatomy and 2Shape exhibited satisfactory results with minimal crack formation.

## Introduction

In endodontic therapy, the prognosis is positively connected with the most effective cleaning and shaping techniques [1]. Nickel-titanium (NiTi) rotary instruments have significantly improved root canal preparation since they have made instrumentation easier and faster [2,3]. Human dentin is viscoelastic, and during biomechanical preparation, instruments contact the root canal wall and apply forces that lead to a temporary stress concentration on the dentin. This may result in minor cracks, which can progress into vertical root fractures during obturation, post-placement, and retreatment [4].

Many variables are out of the control of the clinician (natural root morphology, canal shape, size, and dentin thickness); however, factors that can be addressed during treatment to reduce fracture susceptibility include the final canal shape and extent of canal enlargement [5].

The TruNatomy file system (Dentsply Maillefer, Baillagues, Switzerland) has been recently introduced. The manufacturer claims that this new file system provides the clinician with greater ease, time-saving, safety, enhanced cutting efficiency, and mechanical qualities in comparison to earlier generations of rotary files [6].

After extensive exploration of the available literature, it was found that there is a scarcity of adequate information about the influence of these recently introduced Ni-Ti rotary files in the formation of dentinal cracks. Hence, the objective of the current study was to compare the frequency of crack formation in root dentin following root canal preparation using TruNatomy, Neoendo Flex, ProTaper Gold, and 2Shape using a stereomicroscope. The null hypothesis was that there would be no significant difference in dentinal microcracks produced among the groups.

## Materials and methods

This study was conducted at Government Dental College and Hospital, Patiala, Punjab, India. Eighty extracted single-rooted human permanent mandibular premolars were selected. Each of the chosen teeth was meticulously cleaned and disinfected before being stored in distilled water. The existence of a single canal was confirmed with mesiodistal and buccolingual angulated radiographs. Teeth with fractures, open apices, curved canals, caries or fillings, and complex anatomical variations were excluded from the study. The external root surfaces of the samples were inspected under a stereomicroscope to detect any pre-existing cracks or craze lines.

**Figure 1:**
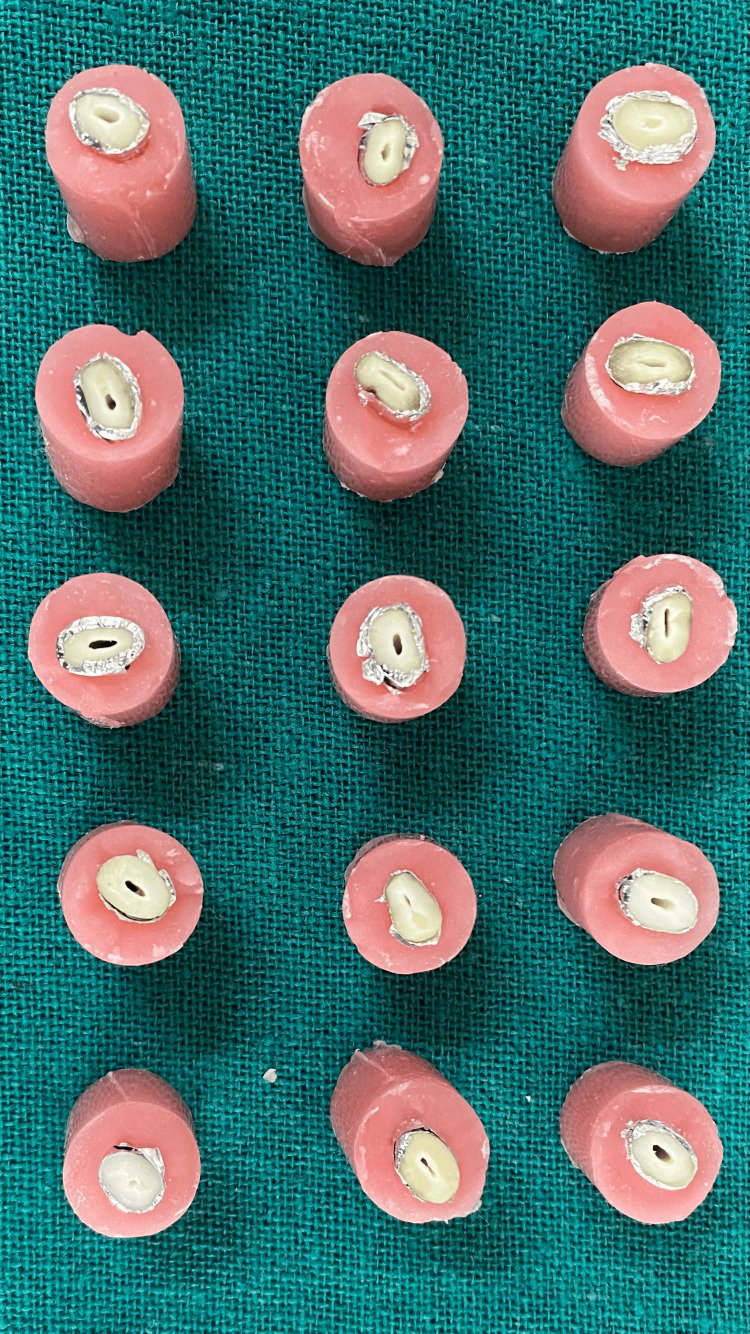
Tooth covered with aluminium foil embedded in self-cure acrylic

**Figure 2:**
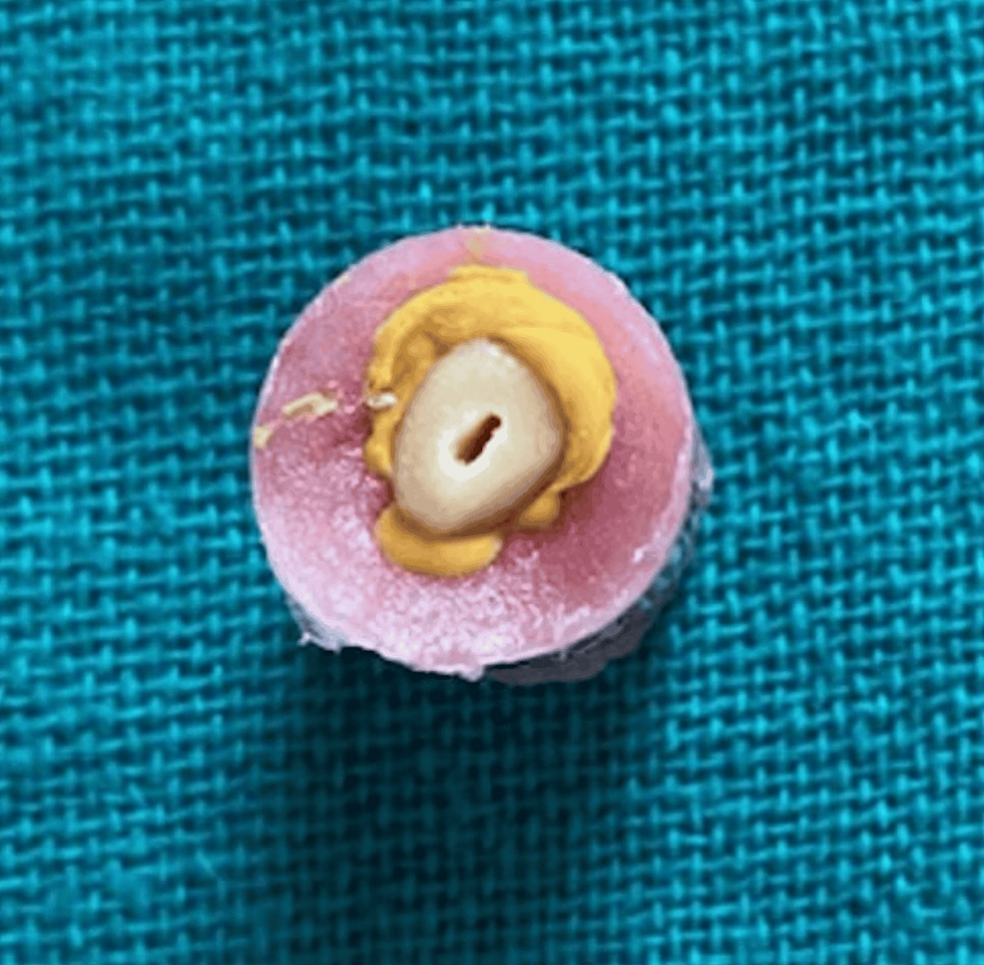
Aluminium foil replaced with a light-body impression material

The teeth were decoronated to obtain a standard length of 16 mm from the apex. Root surfaces were coated with aluminum foil and embedded in self-cure acrylic resin (DPI, India) to simulate bone. Once the acrylic had set, the roots were retrieved. Aluminum foil was removed, and the void it left was filled with light body impression material (GC Flexceed, GC Corporation, Tokyo, Japan) to mimic the periodontal ligament. The roots were then remounted into the acrylic (Figures 1-2).

The patency of root canals was established using a #10k file (Mani Inc., Tokyo, Japan), and the working length was measured by inserting a #10k file into the canal until its tip was visible at the apex and 1 mm subtracted from the initial length. A glide path preparation was done using #15K files. Based on the different Ni-Ti files used, the teeth were randomized into four groups with 20 samples in each: Group I: TruNatomy (Dentsply Maillefer, Baillagues, Switzerland), Group II: Neoendo Flex (Orikam Healthcare, India), Group III: ProTaper Gold (Dentsply Maillefer, Baillagues, Switzerland), and Group IV: 2Shape (MicroMega, Besancon, France). Cleaning and shaping were done in all the teeth from Groups 1 to 4 using the respective rotary file systems until instrument size #25 in all four groups.

The endodomotor (X-Smart, DENTSPLY Tulsa Dental Specialties, Tulsa, USA) was set to a torque and speed of 1.5 Ncm and 300 rpm, respectively, for all the groups. Group I: The canals were prepared using the TRN (Dentsply Maillefer, Baillagues, Switzerland) orifice modifier (20/0.08), TRN glider (17/0.02), and TRN Prime (26/0.04). Group II: The canals were prepared using Neoendo Flex (Orikam Healthcare, India) rotary files sequentially according to the manufacturer’s recommendation to a size of 25 (0.04). Group III: The canals were prepared using PTG (Dentsply Maillefer, Baillagues, Switzerland) starting with shaping file SX (19/0.04), followed by S1 (18/0.02) and S2 (20/0.04). Thereafter, finishing files were used in a sequence of F1 (20/0.07) and F2 (25/0.08) up to the working length. Group IV: The canals were prepared with 2Shape (MicroMega, Besancon, France) rotary files according to the manufacturer’s recommendation using TS1 of size 25 (0.04).

**Figure 3:**
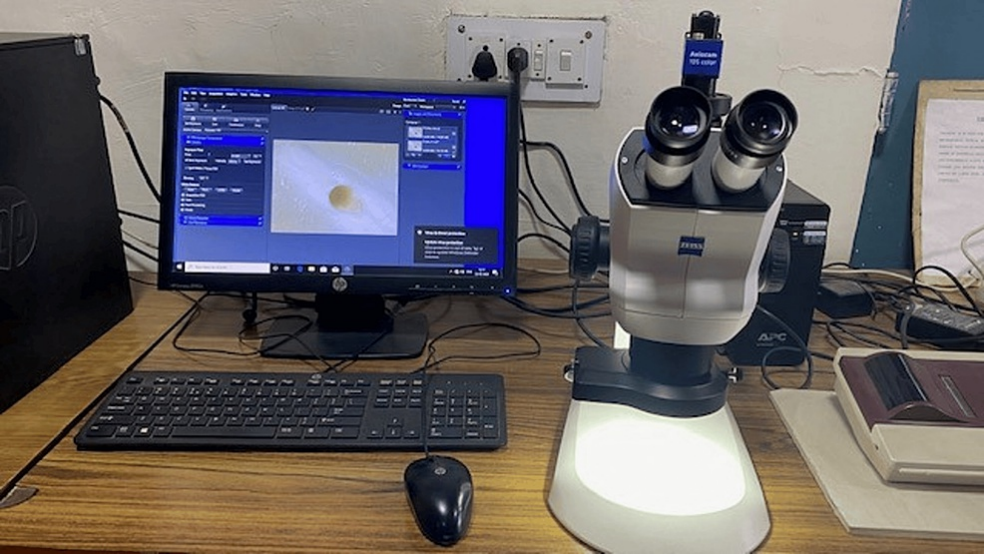
Evaluation of microcracks under a stereomicroscope with an attached camera

The canals were disinfected with 5% sodium hypochlorite (Prevest DenPro Limited, India) and saline using a syringe and 30-gauge side vent needle (Orikam Healthcare, India) between each instrument change. All the samples were kept moist throughout the procedure to prevent dehydration. The roots were then horizontally sectioned at 3 mm (apical) and 6 mm (middle) from the apex using a diamond disc under water coolant. All the slices were viewed under a stereomicroscope at 40x magnification (Zeiss Stemi 508, Carl Zeiss, Jena, Germany) (Figure 3).

**Figure 4:**
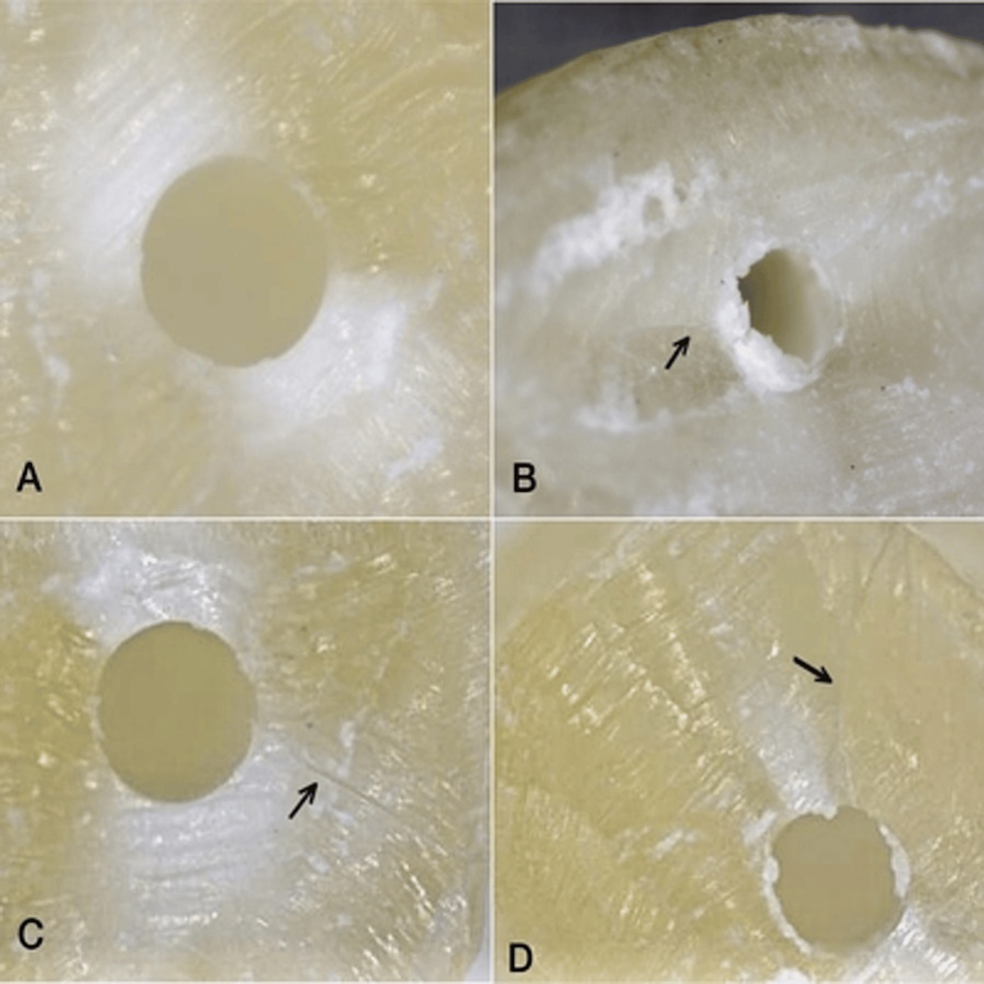
Stereomicroscopic images A: no crack, B: incomplete crack, C & D: complete cracks

A single examiner examined each specimen for microcracks, and images were recorded (Figure 4). The results expressed the number of slices with defects in each group. The technique outlined by Karataş et al. [7] was used to separate dentin cracks into two distinct categories: "No crack" is defined as a root canal dentin without any lines or crack extending from the inner canal wall into the outer dentin. "Crack" includes both complete and incomplete cracks. A "complete crack" is defined as a defect with crack lines extending from the inner root canal space up to the outer surface of the root. An "incomplete crack" is a line that extends from the canal wall into the dentin but does not reach the outer surface.

Statistical analysis

Data were analyzed using the IBM SPSS Statistics for Windows, version 26 (released 2019; IBM Corp., Armonk, New York, United States), and the level of significance was set at p<0.05. Descriptive statistics were performed to assess the mean and standard deviation of the respective groups. Inferential statistics to find out the difference between groups was done using the chi-square test for the proportion.

## Results

Dentinal cracks were observed in all experimental groups. ProTaper Gold resulted in the highest number of cracks (65%), followed by Neoendo Flex (45%), and a lesser number of cracks were seen in TruNatomy (20%) and 2Shape (20%) (Table 1).

**Table 1:** Prevalence of dentin cracks in the experimental groups

	Apical	Middle
Group 1 (TruNatomy)	2 (10%)	2 (10%)
Group 2 (Neoendo Flex)	5 (25%)	4 (20%)
Group 3 (ProTaper Gold)	8 (40%)	5 (25%)
Group 4 (2Shape)	1 (5%)	3 (15%)

The frequency of dentinal cracks in Group 1 (Trunatomy) was 2 (10%), Group 2 (Neoendo Flex) was 5 (25%), Group 3 (ProTaper Gold) was 8 (40%), and Group 4 (2Shape) was 1 (5%). The chi-square analysis showed a statistically significant difference in the frequency of dentinal cracks between the four groups in the apical section (p-value < 0.05) (Table 2).

**Table 2:** Intergroup comparison of the incidence of dentinal cracks (apical section) ^*^p-value <0.05 was considered statistically significant.

	Present	Absent	Chi-square test	p-value
Group 1 (TruNatomy)	2 (10%)	18 (90%)	46.87	0.0001*
Group 2 (Neoendo Flex)	5 (25%)	15 (75%)		
Group 3 (ProTaper Gold)	8 (40%)	12 (60%)		
Group 4 (2Shape)	1 (5%)	19 (95%)		

The frequency of dentinal cracks in Group 1 (TruNatomy) was 2 (10%), Group 2 (Neoendo Flex) 4 (20%), Group 3 (ProTaper Gold) 5 (25%), and Group 4 (2Shape) 3 (15%). The chi-square analysis showed a statistically significant difference in the frequency of dentinal cracks between the four groups in the middle section (p-value < 0.05) (Table 3).

**Table 3:** Intergroup comparison of the incidence of dentinal cracks (middle section) *p-value < 0.05 was considered statistically significant.

	Present	Absent	Chi-square test	p-value
Group 1 (TruNatomy)	2 (10%)	18 (90%)	8.65	0.03*
Group 2 (Neoendo Flex)	4 (20%)	16 (80%)		
Group 3 (ProTaper Gold)	5 (25%)	15 (75%)		
Group 4 (2Shape)	3 (15%)	17 (85%)		

A significant difference in the incidence of microcracks was observed between all the file systems at the apical level except between the TruNatomy and 2Shape groups (p = 0.17) (Table 4), while no significant difference was observed between the files at the middle section except between TruNatomy and PTG (p = 0.005) and TruNatomy and Neoendo Flex (p = 0.04).

**Table 4:** Comparison of dentin cracks between Group 1 and Group 4 (apical section)

	Present	Absent	Chi-square test	p-value
Group 1 (TruNatomy)	2 (10%)	18 (90%)	1.81	0.17
Group 4 (2Shape)	1 (5%)	19 (95%)		

## Discussion

Dentinal cracks can occur when the tensile stress on the root canal wall exceeds the tensile strength of the dentin [8]. These microcracks could propagate under masticatory load, during retreatment, post space preparation, or insertion, and get converted to complete cracks leading to vertical root fractures, often demanding tooth extraction [4]. Kim et al. [9] uncovered a potential link between the design characteristics of NiTi rotary instruments and the frequency of vertical root fractures and concluded that the traits of rotary file design have an impact on the amount of apical stress and strain concentration created during root canal preparation. The geometry of the cross-section, taper, pitch, and flute form are some examples of contributing factors that may be associated with the magnitude of the flaw.

The stress concentration may also be influenced by the complex root canal anatomy, the residual dentinal wall thickness, and the prepared canal diameter. At least 1 mm of sound radicular dentin should be present throughout the length of the root after all intracanal procedures. Excessive removal of the radicular dentin, especially in "danger zones," may result in strip perforation and vertical root fractures. In addition, the gradual dentinal sclerosis caused by the age-related change in the microstructure of dentin may be correlated with decreased resistance to damage initiation and propagation [3,10]. As the thickness of the dentinal wall directly relates to the tooth's resistance to lateral stresses, excessive canal preparation compromises dentinal thickness and weakens the tooth.

Dentinal microcracks were seen in all groups in the current study. In a substantial amount of the treated roots, ranging from 18% to 60% of the roots, all tapered NiTi file systems that have been evaluated so far produce microcracks [11-14].

 ProTaper Gold revealed the greatest number of cracks, while TruNatomy and 2Shape presented the least amount. TruNatomy files have an off-centered parallelogram cross-sectional design, so each time the file rotates in the canal during biomechanical preparation, there is a two-point contact with the root canal wall, thereby engaging less root canal dentin. ProTaper Gold file has a unique convex triangular cross-sectional design, so it establishes three-point contact with the root canal dentinal wall. This facilitates active cutting motion, thereby removing more dentin and at the same time generating more tensile stresses.

According to Kim et al. [9], the stress produced by tapered instruments on the outer surface of dentin may reach values that are higher than the dentin's tensile strength (106 MPa), which ultimately results in the formation of dentinal cracks. According to Wilcox et al. [15], the likelihood of a root fracture rises with the amount of tooth structure lost. The incidence of crack formation and the tapering of the instruments have a significant positive correlation, according to Das et al. [16]. Radicular dentin is more frequently stressed by higher-taper rotary files, which also have a higher likelihood of thinning the remaining dentin. PTG F2 has a larger taper (0.08) compared to the other rotary files used (0.04%), hence removing more radicular dentin and generating greater stress. Bier et al. [12] found cracks in 16% of the roots instrumented with the horizontal sections of the ProTaper system, and they concluded that ProTaper rotary files damaged dentin more than other rotary files. According to Liu et al. [17], 25% of the roots instrumented with the ProTaper had cracks at the apical root surface.

The incidence of dentinal cracks was less with TruNatomy and 2Shape, which are single-file systems, than with PTG and Neoendo Flex, which are multiple-file systems. A single file can be used for both shaping and finishing the canal, rather than instrumenting the canal with a series of files, thereby simplifying the procedure and saving time. According to a study by Jyothilakshmi et al. [18], a single-file system induced fewer defects when compared to multiple-file systems. The rotation of the file in the canal places stresses on the dentin that can lead to microcracks, and this stress increases with the number of files used for biomechanical preparation.

In all the experimental groups, the apical section presented more cracks than the middle sections. This is in line with what Karatas et al. [7], Nishad and Shivamurthy [19], and Chole et al. [20] found in their studies. The root canal systems vary greatly in their cross-sectional anatomy, and root fracture susceptibility may be influenced by the canal morphology. A study conducted by Adorno et al. [11] reported the incidence of apical microcracks in 50% of mandibular premolars after canal preparation to the apical foramen, which is in accordance with the present study. The occurrence of stress due to successive instrumentation, the low capability of the thin and fragile dentin in the apical area to withstand the mechanical stress produced by direct contact with the instrument tip, and features of the files, such as taper angle, flexibility, and cross-section, may also influence the formation of cracks [21].

The possible limitations of the present in vitro study are the possibility of crack formation during sectioning and difficulty in identifying internal preexisting cracks.

## Conclusions

Although the mechanism of vertical root fractures is still not completely understood, it is widely accepted that stresses on the canal wall play a critical role in the initiation of dentinal microcracks. Forces generated during the root canal treatment can be easily controlled by a discerning professional. Meanwhile, masticatory loads are recurrent and cannot be controlled. Even though dentinal cracks are caused by several factors, the most important of which are taper, cross-sectional design, and file flexibility. Preservation of dental hard tissue and maintaining the overall structural integrity of the tooth and tooth root minimize the predisposition to a vertical root fracture after root canal treatment.

Within the limitations of this in vitro study, it can be concluded that rotary NiTi instruments do cause dentinal microcracks during biomechanical preparation. ProTaper Gold and Neoendo Flex induced significantly more dentinal cracks than TruNatomy and 2Shape at 3 mm and 6 mm levels. The maximum number of dentinal defects was seen in the apical third region. According to this in vitro study, single-file systems resulted in a lesser number of cracks compared to multiple-file systems.

## References

[1] Kesim B, Sagsen B, Aslan T (2017). Evaluation of dentinal defects during root canal preparation using thermomechanically processed nickel-titanium files. Eur J Dent.

[2] Hashem AA, Ghoneim AG, Lutfy RA, Foda MY, Omar GA (2012). Geometric analysis of root canals prepared by four rotary NiTi shaping systems. J Endod.

[3] Panda A, Shah K, Budakoti V, Dere K, Virda M, Jani J (2021). Evaluation of microcrack formation during root canal preparation using hand, rotary files and self-adjusting file in primary teeth: an in vitro study. J Dent Res Dent Clin Dent Prospects.

[4] Bhavsar BA, Sharma P, Surana P, Badnaware S, Jadhaw D, Jain A (2023). Oval root canals prepared with two different endodontic rotary files: an in vitro study comparing the incidence of dental defects. Cureus.

[5] Versluis A, Messer HH, Pintado MR (2006). Changes in compaction stress distributions in roots resulting from canal preparation. Int Endod J.

[6] van der Vyver PJ, Jonker C (2014). Reciprocating instruments in endodontics: a review of the literature. S Afr Dent J.

[7] Karataş E, Gündüz HA, Kırıcı DÖ, Arslan H, Topçu MÇ, Yeter KY (2015). Dentinal crack formation during root canal preparations by the twisted file adaptive, ProTaper Next, ProTaper Universal, and WaveOne instruments. J Endod.

[8] Al-Zaka I (2012). The effects of canal preparation by different NiTi rotary instruments and reciprocating WaveOne file on the incidence of dentinal defects. M Dent J.

[9] Kim HC, Lee MH, Yum J, Versluis A, Lee CJ, Kim BM (2010). Potential relationship between design of nickel-titanium rotary instruments and vertical root fracture. J Endod.

[10] Saha SG, Vijaywargiya N, Saxena D, Saha MK, Bharadwaj A, Dubey S (2017). Evaluation of the incidence of microcracks caused by Mtwo and ProTaper Next rotary file systems versus the self-adjusting file: a scanning electron microscopic study. J Conserv Dent.

[11] Adorno CG, Yoshioka T, Suda H (2010). The effect of working length and root canal preparation technique on crack development in the apical root canal wall. Int Endod J.

[12] Bier CA, Shemesh H, Tanomaru-Filho M, Wesselink PR, Wu MK (2009). The ability of different nickel-titanium rotary instruments to induce dentinal damage during canal preparation. J Endod.

[13] Yoldas O, Yilmaz S, Atakan G, Kuden C, Kasan Z (2012). Dentinal microcrack formation during root canal preparations by different NiTi rotary instruments and the self-adjusting file. J Endod.

[14] Shemesh H, Bier CA, Wu MK, Tanomaru-Filho M, Wesselink PR (2009). The effects of canal preparation and filling on the incidence of dentinal defects. Int Endod J.

[15] Wilcox LR, Roskelley C, Sutton T (1997). The relationship of root canal enlargement to finger-spreader induced vertical root fracture. J Endod.

[16] Das S, Pradhan PK, Lata S, Sinha SP (2018). Comparative evaluation of dentinal crack formation after root canal preparation using ProTaper Next, OneShape, and Hyflex EDM. J Conserv Dent.

[17] Liu R, Kaiwar A, Shemesh H, Wesselink PR, Hou B, Wu MK (2013). Incidence of apical root cracks and apical dentinal detachments after canal preparation with hand and rotary files at different instrumentation lengths. J Endod.

[18] B J, Pillai R, Varghese N (2020). Influence of metallurgy and file design in micro crack formation in root canals - an in vitro study. Glob J Med Res.

[19] Nishad SV, Shivamurthy GB (2018). Comparative analysis of apical root crack propagation after root canal preparation at different instrumentation lengths using Protaper Universal, Protaper Next and Protaper Gold rotary files: an in vitro study. Contemp Clin Dent.

[20] Chole D, Kamble S, Bakle S, Gandhi N, Hatte N, Bawa P (2019). Effects of 3 single-file systems (Protaper Universal, Protaper Next, Protaper Gold) on crack formation in dentin after root canal preparation - an in-vitro study. IOSR J Dent Med Sci.

[21] Tomer AK, Ramachandran M, John AG, Jose TK, Tirupathi P, Kumari S, Raina A (2019). Evaluation of dentinal crack formation during root canal preparation using protaper gold, Mani silk files- in vitro study. Int J Appl Dent Sci.

